# A Novel Case of Yamaguchi Syndrome in a Hispanic Male

**DOI:** 10.7759/cureus.17651

**Published:** 2021-09-01

**Authors:** Farhan A Shah, Priscilla Fujikawa, Jeremy B Miller, Harleen Singh

**Affiliations:** 1 Internal Medicine, LewisGale Medical Center, Salem, USA; 2 Internal Medicine, Edward Via College of Osteopathic Medicine-Virginia, Blacksburg, USA

**Keywords:** yamaguchi, apical hypertrophy, apical cardiomyopathy, ace of spades, hypertrophic cardiomyopathy

## Abstract

Yamaguchi syndrome or apical hypertrophic cardiomyopathy (HCM) is a unique variant of HCM. It is characterized by localized hypertrophy involving the left ventricular apex rather than the left ventricular septum. This syndrome has been traditionally seen in the Asian population, particularly those of Japanese descent. We present an interesting case of Yamaguchi syndrome seen in a Hispanic male.

A 48-year-old Hispanic male presented with epigastric tenderness and was admitted to the hospital for a non-ST-segment elevation myocardial infarction. His diagnostic catheterization revealed no significant coronary artery disease. However, his echocardiogram revealed apical hypertrophy and narrowing of the left ventricular cavity at the apex, consistent with Yamaguchi syndrome. Case reports such as ours serve to help clinicians broaden their differential diagnoses when approaching patients with acute coronary syndrome-like symptoms to include diagnoses such as Yamaguchi syndrome.

## Introduction

Yamaguchi syndrome, also known as apical hypertrophic cardiomyopathy (HCM), is a rare form of nonobstructive HCM. First described in 1976 by H. Yamaguchi, it was presumed to be confined to the Japanese population with a prevalence of 15-25% in the Asian population. It is now known to be found in other populations as well with a prevalence of almost 3% in the United States. Unlike HCM, which predominates in the left ventricular septum, apical HCM tends to involve the left ventricular apex. Apical HCM often mimics acute coronary syndrome (ACS), causing the diagnosis to be frequently missed or delayed. It has a wide array of clinical manifestations including, but not limited to, chest pain, palpitation, dyspnea, syncope, and heart failure. Management of this disease often includes symptomatic treatment. Prognosis of the apical form of HCM is relatively benign with a lower mortality rate than other forms of HCM. However, approximately one-third of patients can develop serious, life-threatening complications, and thus close follow-up is warranted [1].

## Case presentation

A 48-year-old Hispanic male with no significant medical history presented to the emergency department complaining of epigastric tenderness. He stated that pain initially began in his mid-thoracic back region and radiated to his epigastrium. The pain had lasted for one week, was aggravated with any positional changes, and was alleviated with rest. He denied chest pain with exertion, shortness of breath, or palpitations. He was a painter by profession and had not had any difficulty performing his job. He was a nonsmoker and did not consume alcohol. He reported that he was told that he had hypertension in the past but was not taking any medications.

**Figure 1:**
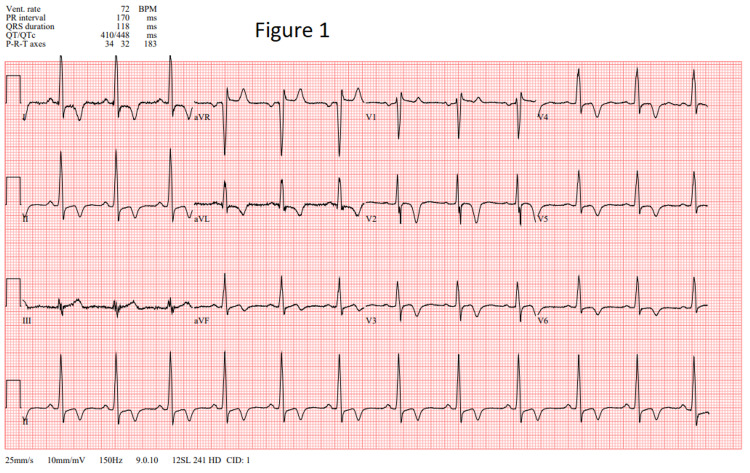
EKG demonstrating characteristic T wave inversions. EKG: electrocardiogram

In the emergency department, his physical examination revealed normal vital signs, including normotension, without any other acute findings, except mild point tenderness in the epigastrium. Laboratory findings were unremarkable except for an elevated troponin of 0.074 ng/mL (reference: 0.000-0.045 ng/mL). Electrocardiogram (EKG) revealed T wave inversions in leads I, II, and V2-V6 (Figure 1). The Thrombolysis in Myocardial Infarction score was 2. The chest radiograph did not reveal any acute process. The patient received full-dose aspirin and was admitted for non-ST-segment elevation myocardial infarction.

After admission, he was started on full-dose enoxaparin sodium, metoprolol tartrate, and lisinopril. He could not tolerate his antihypertensive regimen as he remained hypotensive with systolic blood pressure in the range of 90-100 mmHg and bradycardic in the range of 40-50 beats per minute while he remained hospitalized. His affecting medications were discontinued as a result.

**Figure 2:**
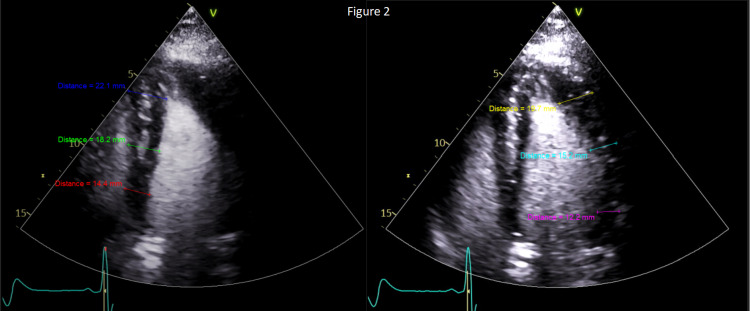
Echocardiogram with ventricular wall measurements and classic “ace of spades” shape. Left image: Transthoracic two-chamber view of the left ventricle and atrium, with free wall on the right side of the image. Contrasted with perflutren (lipid microsphere contrast agent). Right image: Transthoracic four-chamber view using perflutren. Left ventricle free wall to the right side of the image.

His troponin peaked at 0.083 ng/mL and trended downward to 0.077 ng/mL. He underwent left heart catheterization to evaluate for occlusive coronary disease. The diagnostic catheterization revealed no significant coronary artery disease angiographically to explain the patient’s symptoms. However, his echocardiogram revealed apical hypertrophy and narrowing of the left ventricular cavity at the apex, resulting in an “ace of spades” morphology, characteristic of Yamaguchi syndrome or apical HCM (Figure 2). His EKG findings and clinical presentation were attributed to his long-standing cardiomyopathy and not due to ACS. Once medically stable, the patient was discharged.

## Discussion

Apical HCM was first described by Dr. Yamaguchi et al. in a 1979 article based on 1,002 consecutive left heart catheterization patients. Of the total, 30 patients exhibited the now pathognomonic “ace of spades” shape of the left ventricle (LV) [2]. Subsequent studies have shown a predominance of incidence in the Asian population compared to the American population. A comparison by Kitaoka et al. showed a prevalence of 15% in an unselected Japanese cohort as opposed to 3% in an unselected Minneapolis cohort. The same comparison showed that incidence among men is significantly higher than that among women, regardless of Japanese versus American nationality [3]. A German study examining over five million patients showed similar results, specifically that in all age groups men had a higher prevalence than women; additionally, the incidence increased with age and further favored men as age increased [4]. Based on the literature review, there is a dearth of information regarding incidence in the African American/Black population, with only one case report available [5]. We were unable to find a case study or population study regarding incidence among Hispanic patients.

Apical HCM has a male predominance with a male-to-female ratio of approximately 1.6 to 2.8:1. The average age of presentation is 41.4 + 14.5 years. Most patients with apical HCM present with mild or no symptoms. The most common complaints are angina, atypical chest pain, shortness of breath, palpitations, dyspnea, and presyncopal/syncopal episodes. On physical examination, audible and palpable fourth heart sound can be heard, reflecting impaired LV relaxation. Almost half of the patients with apical HCM have abnormalities in the mitral valve, similar to those of HCM. This can lead to systolic anterior motion of the mitral valve and mitral regurgitation findings on the examination as well [1].

Several phenotypic mimics of apical HCM exist. Apical displacement of papillary muscle (ADPM) can mimic apical HCM, and differentiating the two can be challenging. Physical examination and symptoms can be similar between the two, and EKG findings of apical HCM (left ventricular hypertrophy, T wave inversion of 10 mm) may be present in ADPM. In apical HCM complicated by left ventricular outflow obstruction, auscultation may reveal a crescendo-decrescendo systolic murmur in the left sternal border due to turbulence within the LV and mitral incompetence. Therefore, careful multimodality imaging is important to differentiate the two [6].

Chest pain in patients with apical HCM can be mistaken for ischemia due to coronary artery disease. Often, a nuclear scan is performed for these patients with abnormal EKGs. The majority of patients with apical HCM who have a myocardial infarction have it in the apical region. Thus, wall motion abnormalities can vary from apical aneurysm to apical hypokinesis. Therefore, apical aneurysms can be seen in patients with asymptomatic apical HCM [7].

Other diseases included in the differential diagnosis of apical HCM include apical cardiac tumors, isolated ventricular compaction, LV apical thrombus, endomyocardial fibrosis, cardiac tumors, long-term athletic training (“athlete’s heart”), and coronary artery disease [7].

Several complications can occur in patients with apical HCM. In a retrospective study of 105 patients with apical HCM diagnosed at the Toronto General Hospital from 1975 to 2000, it was found that one-third of apical HCM patients may develop potentially life-threatening complications. The most common complications were atrial fibrillation (12%) and myocardial infarction (10%). Other complications of apical HCM include apical aneurysms and cardiac arrest. Overall survival in this subset was 95% at 15 years, and the probability of survival without morbid events was 74% at 15 years. Predictors of morbidity in apical HCM were identified as follows: age at presentation <41 years, left atrial enlargement, and New York Heart Association (NYHA) class ≥II at baseline [8].

Apical HCM is reported to have an autosomal dominant inheritance pattern with most mutations located in the sarcomere. The majority of the gene mutations occur in *myosin‐binding protein C* (*MYBPC3*) and *β‐myosin heavy chain* (*MYH7*) in the thick myofilaments of the cardiac tissue [9]. One study using a nine-gene panel showed that 25% of 71 apical HCM versus 34% of 1,053 all subtypes of HCM patients had genetic defects. These genes included *cardiac α‐actin 1* (*CTC1*), *MYBPC3*, *MYH7*, *myosin regulatory light chain* (*MYL2*), *myosin essential light chain* (*MYL3*), *cardiac troponin T2* (*TNNT2*), *cardiac troponin I3* (*TNNI3*), *troponin C1, slow skeletal and cardiac type* (*TNNC1*), and *α‐tropomyosin 1* (*TPM1*) [10]. There are also three metabolic cardiomyopathy genes associated with apical HCM: *α‐galactosidase A* (*GLA*) for Fabry disease; *lysosomal-associated membrane protein‐2* (*LAMP2*) for Danon disease; and *protein kinase, AMP‐activated, noncatalytic, gamma‐2* (*PRKAG2*) for PRKAG2 cardiomyopathy. Studies have also reduced mutation rates in apical HCM compared to HCM (13% versus 40%, respectively, with an eight-gene panel) [9,10].

Unlike HCM, apical HCM has fewer patients reporting a positive family history. This suggests that genetics and environmental factors potentially play a role in the disease. However, in a study comparing apical HCM genotype versus phenotype correlations, those with sarcomere gene mutations, in general, had a stronger correlation with a positive family history of HCM [5]. Thus far, there has been no evidence suggesting a difference in the clinical course of apical HCM genotype-positive individuals versus negative individuals [1,9].

If a patient presents a specific genetic mutation associated with apical HCM, genetic screening is recommended in first-degree relatives. Genotype-positive and phenotype-negative family members should be evaluated by EKG and echocardiography every three to four years for adults and 12-18 months for children. Genetic testing in apical HCM is relatively low with only 25% positivity; however, this leaves room for growth in identifying mechanisms associated with apical HCM [10].

The diagnosis of apical HCM can be achieved by multiple different modalities. The key EKG finding is the “giant negative T waves” (greater than or equal to 10 mm) in the precordial leads and high QRS voltage in the absence of notable coronary artery disease [8,10,11]. These deep, inverted T waves are often mistaken for ACS [12]. A recent study from the Mayo Clinic reported that only 11% of patients, rather than the previously thought 50%, had giant T waves [10]. Other studies have shown that deep T wave inversions are commonly absent in the non-Japanese population [8]. Patients who lacked giant negative T waves had apical hypertrophy localized to the most distal area of the septum or the anterolateral portion of the apex. On the other hand, patients whose EKGs revealed giant inverted T waves had diffuse hypertrophy of the apex [8].

On the echocardiogram, an asymmetrical left ventricular wall thickness greater than 15 mm during diastole is diagnostic of apical HCM [8,10,12]. A lower cutoff of 13 mm can be used if the clinical scenario, imaging, and family history lead to the same diagnosis. A ratio of apical to posterior wall thickness of 1.5 or greater is also consistent with apical HCM [8]. A unique characteristic of apical HCM seen on the echocardiogram is midventricular obstruction and cavity obliteration during systole. When this feature progresses into diastole and a paradoxical jet flow occurs from the apex to the base, it indicates severe disease and worse prognosis. This occurs because the phenomenon is associated with a higher risk of systemic embolism, perfusion abnormalities, and ventricular arrhythmias [10]. It is important to note that the echocardiogram can provide limited information depending on the quality of the echocardiographic view [13].

The cardiac MRI can be a more reliable tool and is recommended by the American Heart Association guidelines to aid in risk stratification when sudden cardiac death (SCD) risk stratification is ambiguous using standard methods [10,13]. Cardiac MRI in apical HCM reveals late gadolinium enhancement (LGE), consistent with dense extracellular matrix deposition and fibrosis, which is thought to be a result of multiple episodes of microvascular ischemia. LGE occurs in up to 75% of individuals affected by HCM. An LGE of greater than or equal to 15% of the left ventricular volume is associated with double the risk of SCD [10]. A wall thickness greater than or equal to 30 mm is associated with a critical risk of SCD [8].

A left ventriculogram in the right anterior oblique position allows for the visualization of the “spade-like” shape (as in the ace of spades) at the end of diastole, consistent with apical hypertrophy and distal cavity obliteration during systole [8,12,14].

Apical HCM is managed medically in most cases. Although studies are scarce in this field, beta-blockers are believed to be the gold standard. No studies have been done to compare the different types of beta-blockers, but the beta-1 selective subclass is preferred to avoid worsening of the midventricular gradient. They decrease the chronotropic response of the heart, especially during exercise, thus reducing myocardial oxygen demand and maximizing diastolic filling. The long-term benefits of beta-blockers are unknown [10]. There is contradicting evidence regarding the benefits of angiotensin-converting enzyme inhibitors and angiotensin II receptor blockers in patients with HCM, although angiotensin-converting enzyme inhibitors could potentially reduce cardiac remodeling and fibrosis seen in apical HCM [10,12]. Small studies, such as the Ranolazine for Treatment of Angina or Dyspnea in Hypertrophic Cardiomyopathy Patients (RHYME) and the Effect of Eleclazine on Exercise Capacity in Subjects With Symptomatic Hypertrophic Cardiomyopathy (LIBERTY-HCM), have begun to evaluate the potential of late sodium current inhibitors in patients with hypertrophic cardiomyopathy [10]. However, more extensive research is warranted. Alcohol ablation is considered in patients with midventricular obstruction who are at high risk for surgery for symptomatic management of heart failure [10].

Patients who have persistent symptoms and remain an NYHA class 3-4 despite optimal medical therapy are considered for surgical interventions as a bridge to heart transplantation. Options include an extended myectomy with ventricular reconstruction or an apical myectomy. Apical myectomy is a new technique that involves cardiopulmonary bypass and aims to enlarge the LV allowing for a higher end-diastolic volume. The ideal wall thickness at the end of the procedure is 10-12 mm. Overall, 70% of patients who underwent apical myectomy at the Mayo Clinic from 1993 to 2012 improved to an NYHA class 1-2 [10].

Patients who have apical HCM with apical aneurysm and who develop sustained ventricular tachycardia (VT) are difficult to manage medically. VTs frequently recur and progress into ventricular fibrillation leading to refractory cardiogenic shock. Implantable cardioverter-defibrillators can be placed to prevent sudden cardiac death; however, they cannot avoid refractory VT. Catheter ablation is also challenging due to the fibrotic myocardium and the narrow aneurysm neck. Therefore, surgical ablation with electroanatomic mapping is the most appropriate alternative for accurate targeting of the arrhythmogenic source, management of sustained ventricular tachycardia, and prevention of progressive LV dysfunction [10]. Similarly, ventricular-assist devices are also considered a bridge to transplant, which is therapeutic and crucial, especially in patients with refractory life-threatening arrhythmias. A large study from the United Network of Organ Sharing Registry showed that HCM and non-HCM patients share similar rates of survival following heart transplant: 85% at one year, 75% at five years, and 61% at ten years [10].

Patients with apical HCM should avoid strenuous physical activity. Most people are able to continue their regular job without restrictions. Genetic counseling should be discussed with women of child-bearing age as well as the inheritance patterns of this disease. In the majority of cases, females are able to carry a pregnancy without an increase in filling pressures but a thorough pre-pregnancy risk assessment should be performed [10].

Apical HCM has an overall benign long-term prognosis with an annual mortality of 0.1%, which is lower than the 1.4-4% annual mortality of all types of hypertrophic cardiomyopathy [12,13]. Characteristics associated with a poor prognosis include young age at diagnosis, family history of sudden cardiac death, and NYHA class II or greater [12]. Patients should be followed up annually for repeat echocardiograms [11].

## Conclusions

Although Yamaguchi syndrome is traditionally seen in the Asian, particularly Japanese, populations, this case report highlights that it can be seen in the Hispanic population as well. Clinicians should consider Yamaguchi syndrome as a possible differential when evaluating patients with angina-like symptoms.
